# Valorization of Spent Coffee Grounds as Precursors for Biopolymers and Composite Production

**DOI:** 10.3390/polym14030437

**Published:** 2022-01-22

**Authors:** Anne Shayene Campos de Bomfim, Daniel Magalhães de Oliveira, Herman Jacobus Cornelis Voorwald, Kelly Cristina Coelho de Carvalho Benini, Marie-Josée Dumont, Denis Rodrigue

**Affiliations:** 1 Fatigue and Aeronautical Materials Research Group, Department of Materials and Technology, UNESP-São Paulo State University, Guaratinguetá 12516-410, São Paulo, Brazil; anne.shayene@unesp.br (A.S.C.d.B.); daniel.m.oliveira@unesp.br (D.M.d.O.); h.voorwald@unesp.br (H.J.C.V.); kcccbenini@gmail.com (K.C.C.d.C.B.); 2 Bioresource Engineering Department, McGill University, 21111 Lakeshore Road, Ste-Anne-de-Bellevue, QC H9X 3V9, Canada; marie-josee.dumont@mcgill.ca; 3 Department of Chemical Engineering and CERMA, Université Laval, Quebec, QC G1V0A6, Canada

**Keywords:** spent coffee grounds, biopolymer precursors, polysaccharides, composites

## Abstract

Spent coffee grounds (SCG) are a current subject in many works since coffee is the second most consumed beverage worldwide; however, coffee generates a high amount of waste (SCG) and can cause environmental problems if not discarded properly. Therefore, several studies on SCG valorization have been published, highlighting its waste as a valuable resource for different applications, such as biofuel, energy, biopolymer precursors, and composite production. This review provides an overview of the works using SCG as biopolymer precursors and for polymer composite production. SCG are rich in carbohydrates, lipids, proteins, and minerals. In particular, carbohydrates (polysaccharides) can be extracted and fermented to synthesize lactic acid, succinic acid, or polyhydroxyalkanoate (PHA). On the other hand, it is possible to extract the coffee oil and to synthesize PHA from lipids. Moreover, SCG have been successfully used as a filler for composite production using different polymer matrices. The results show the reasonable mechanical, thermal, and rheological properties of SCG to support their applications, from food packaging to the automotive industry.

## 1. Introduction

Coffee, as a beverage, is consumed all over the world and is now considered a commodity. In 2018, coffee production was evaluated at 9.5 million tons [1,2]. After brewing, SCG are generated as waste, corresponding to about 90% of the initial coffee beans [3]. Thus, SCG waste is an environmental concern, since it can be toxic when not properly discarded because of the caffeine, tannin, and polyphenol emissions during coffee fermentation [4]. This is why efforts to valorize and reuse this waste via environmentally friendly pathways, ones that do not cause any kind of pollution, have been proposed. SCG, which are a lignocellulose-rich waste, have been applied in different fields, such as biofuel production, energy production, and in polymer precursors and composites [5,6,7].

**Figure 1:**
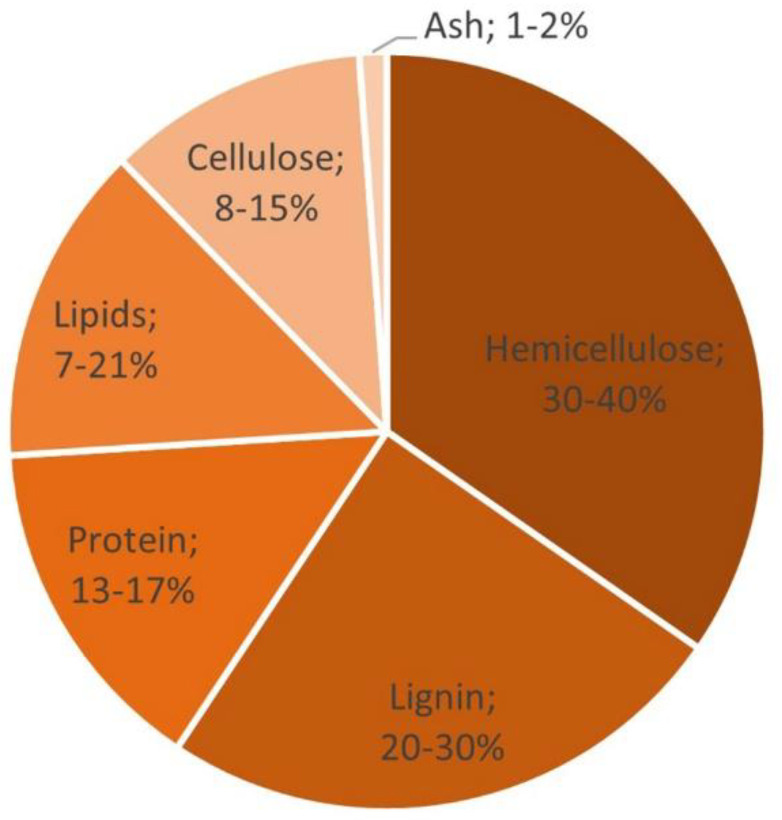
SCG chemical compounds after brewing.

It is well established that SCG are rich in carbohydrates, lipids, proteins, and minerals. Much attention has been given to the extraction and valorization of the individual fractions of SCG. Carbohydrates represent half of the total coffee beans, being mainly polysaccharides of hemicellulose (30–40 wt.%) and cellulose (8–15 wt.%) [8,9] (Figure 1). These polysaccharides can be hydrolyzed to obtain fermentable sugars, such as mannose, glucose, galactose, and arabinose, which can then be converted by microbial fermentation into lactic acid, acetic acid, succinic acid, polyhydroxyalkanoate (PHA), and other molecules of interest [10,11,12]. The SCG oil fraction (7–21 wt.%) is also a valuable resource that is reported in PHA synthesis, biosurfactant production, biodiesel and bioethanol synthesis, as well as in sunscreen formulations [13,14]. The residues of SCG can be directly used as fillers in composites (mainly polymer matrices), or after chemical modifications (the extraction of the molecules of interest). Typical examples of polymer matrices are synthetic polymers, such as polyurethane [15] and polypropylene [16,17,18], or biopolymers, such as polylactic acid (PLA) [19,20,21]. The resulting materials have been proposed for various applications, such as for packaging (mostly food), disposable products (single-use products/composting), and 3D printing [15,21,22]. The development of SCG composites is proposed as a sustainable pathway because it reduces the amount of SCG waste in the environment [22] while creating added-value products, which can even accelerate the degradation process of a biodegradable matrix [23].

Several reviews report the possible valorization of SCG and other coffee byproducts [2,3,8,11,24]. Nevertheless, none of them discuss in detail, with specific applications, their use in the plastics industry. This work aims to fill this gap by presenting the state-of-the-art possibilities of SCG valorization in the plastics field over the last 20 years (2000–2021). In particular, there is a focus on polymer precursors, biopolymer production, and composites. The first part reports information on the composition of SCG, the possibility of using SCG as green polymer precursors, and their application in different polymeric matrices. Then, future prospects are discussed, highlighting the significance of this subject for more academic/scientific research, as well as for commercial/industrial developments.

## 2. Polysaccharide Extraction from SCG

Various polysaccharides can be extracted from SCG. As stated, these polysaccharides mainly consist of hemicelluloses, such as galactomannan and arabinogalactan, as well as cellulose, galactan, mannan, and arabinan, in lower amounts [25,26,27]. Mussatto et al. (2011) identified a hemicellulose composition with mannose as the main monosaccharide present (21.2 g/100 g), followed by galactose (13.8 g/100 g), and arabinose (1.7 g/100 g); however, no xylose was found [25].

Alkali extraction is the main technique used to extract polysaccharides from SCG, and it allows access to the molecules still present after infusion. The technique consists of soaking the SCG in hot water, followed by a series of soakings at room temperature with the progressive addition of a base, such as potassium hydroxide or sodium hydroxide, which leads to the precipitation of polysaccharides [26,27,28]. The extraction sequence was: 2 L of distilled water (90 °C for 1 h), followed by 2 L and 0.5 M of imidazole (70 °C for 1 h) and 1-L NaOH extractions (0.05, 1, and 4 M), three times at room temperature for 2 h; however, an inert atmosphere is required for the last extractions to avoid alkaline oxidation and the peeling reactions of the polysaccharides [26]. The extract can then be filtered, followed by the suspension and dialysis of the precipitate containing the polysaccharides, followed by centrifugation. The defatting of the SCG can be considered prior to alkali extraction [29]. It is also possible to do a roasting pretreatment, leading to an increase in the polysaccharide extraction yield [30]. Following their extraction, the polysaccharides can be hydrolyzed to separate the monosaccharides by enzymatic or acid hydrolyses [31]. It should be noted that hydrolysis via the anaerobic digestion of polysaccharides is also possible [32]. Another method of extracting the polysaccharides from SCG is through the application of an alkali extraction with hydrogen and peroxide in the solution [33]. Finally, dilute acid hydrolysis is another option [25]. The main advantage of the latter method is that the monosaccharides forming the polysaccharides are released and separated, thus avoiding the need of the extra hydrolysis step for the other extraction methods. Dilute acid hydrolysis consists of soaking the SCG in sulfuric acid at a high temperature (100–180 °C) in a batch reactor. At the end of the reaction, the reactor is cooled down in an ice bath, and the solid material can be separated by filtration, with the filtrate containing the polysaccharide hydrolysates. The optimal conditions for hydrolysis were found to be at 100 mg of acid/g of dry matter, and a 10 g/g liquid-to-solid ratio at 163 °C for 45 min. Following the hydrolysis step, various compounds are formed, including hydroxymethylfurfural (HMF), acetic acid, phenols, and heavy metals, which interfere with the bioconversion processes, e.g., the fermentation of the hydrolysate, and inhibit them [34]. To purify and separate the hydrolysate from the compounds of these inhibitors, different methods can be used, such as biological methods, involving enzymes; physical methods, involving the evaporation of the volatile compounds; and chemical methods, involving precipitation or ionization. These methods can be used alone or in any combination.

Autohydrolysis is a new method proposed for the extraction of polysaccharides that does not require any chemical agent, as it only involves water. In an autohydrolysis process, SCG are mixed with water at a very high temperature (160–200 °C) in a batch reactor. The temperature, the solid-to-liquid ratio, and the extraction time play crucial roles in the extraction performance, and the optimal conditions are 160 °C, 15 mL/g of SCG, and 10 min, respectively. Following extraction, the reactor is cooled down in an ice bath. The liquid phase is then mixed with ethanol to precipitate the polysaccharides, which can then be recovered by centrifugation [5,35].

It should be noted that pretreatments can be performed on the SCG before the polysaccharide extraction via water hydrolysis, ultrasound, microwave, or supercritical carbon dioxide (SC-CO_2_). Some parameters can be set to compare the yields of the SCG after each pretreatment, such as the extraction temperature, pressure, and time. The optimal conditions were found to be 180 °C, 40 bar, and 10 min, respectively. On the basis of these results, it was found that the ultrasound pretreatment led to higher yields (18 wt.%) and it was shown to be a more successful method for extracting polysaccharides from SCG [36]. The microwave-assisted pretreatment can work at a lower operation time and is influenced by the temperature, the microwave radiation exposure time, and the water or alkali solution in the suspended sample. The microwave pretreatment followed by water hydrolysis showed that arabinogalactan needs a higher temperature to be extracted (up to 170 °C), while galactomannan can be extracted at lower temperatures (from 140 °C) [37,38].

## 3. Conversion of Polysaccharides and Their Hydrolysates into Biopolymer Precursors

### 3.1. 2,3-Butanediol Synthesis

**Figure 2:**
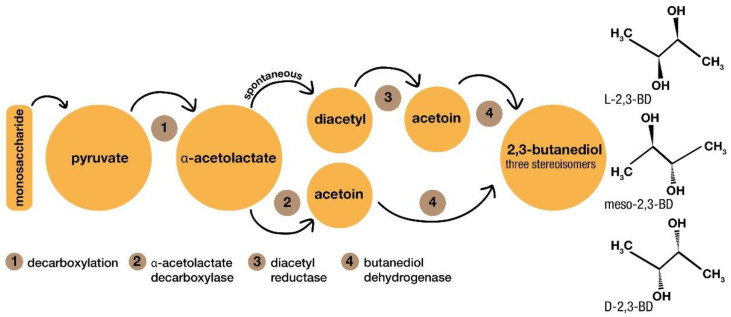
The synthesis steps leading to 2,3-butanediol.

Butanediol (BD) is a chemical compound obtained via microbiological fermentation, with an alternative bioprocess for several industrial applications, such as polyesters, rubbers, fertilizers, cosmetics, pharmaceuticals, and food additives [39]. BD is synthesized by different microorganisms in a mixed acid fermentation process that produces other coproducts, such as ethanol, acetoin, acetate, lactate, and succinate, depending on the microorganism used, the pH value, and the oxygen level. The synthesis of 2,3-BD occurs from pyruvate with three main enzymes (α-acetolactate synthase, α-acetolactate decarboxylase, and butanediol dehydrogenase), as reported in Figure 2 [40]. Pyruvate is generated from some monosaccharides, such as glucose, xylose, or other sugars. From glucose, pyruvate is produced by glycolysis. It is then converted to α-acetolactate via decarboxylation, followed by acetoin formation via α-acetolactate decarboxylase. In the presence of oxygen, acetoin can also be formed by diacetyl reductase after a spontaneous formation of diacetyl. Both types of acetoin are converted to 2,3-BD via butanediol dehydrogenase. It is important to know that 2,3-BD can be formed with different isomers because of the distinct acetoin formation [40,41,42].

The oxygen supply, the pH, and the temperature should be considered when starting the fermentation process, which is different between laboratory and industrial conditions. The yield of the 2,3-BD production, and the effectiveness of the aerobic conditions, are mostly related to the oxygen level. An appropriate initial pH (6–7) and pH setting (5–8) during the whole fermentation process directly affect the production. Moreover, bacteria inoculums only grow under specific temperatures (30–37 °C), but selecting an appropriate temperature directly affects the yield of 2,3-BD. After the fermentation process, 2,3-BD purification should be evaluated and optimized, but its high boiling point (180–184 °C) and hydrophilic behavior make the separation and purification more difficult [42,43,44].

Despite the biological and nontoxic process, the yield and the cost of 2,3-BD synthesis is a current issue that stands against the use of petroleum-based chemicals. This is why there have been some attempts to enhance 2,3-BD synthesis, in terms of higher yields, lower costs, and an efficient substrate feedstock [39]. Therefore, waste materials rich in lignocellulose are attractive for 2,3-BD production because they are inexpensive, abundant, and nontoxic. In the last few years, lignocellulosic biomass has been used as a substrate for 2,3-BD production, since cellulose and hemicellulose can be hydrolyzed to recover 2,3-BD [42,45]. Several works present good results using microorganisms and lignocellulosic biomasses to produce 2,3-BD (Table 1). Moreover, Hazeena et al. (2020) report a list of lignocellulosic biomasses that have already been used in the literature for 2,3-BD production, e.g., corn stover, corncobs, sugarcane bagasse, rice, kenaf, and soybean hull [46].

There is no work on the direct production of 2,3-BD from SCG. However, it has been reported that 2,3-BD was detected after the fermentation of green coffee beans from *C. cellulans*, with a spontaneous fermentation using mesophilic and lactic acid bacteria to improve the fermentation process [47], and from *Rhizopus oligosporus,* with valine biosynthetic fermentation, followed by diacetyl formation and acetoin formation aimed at the modulation of the coffee aroma [48]. These two works detected 2,3-BD, but this was not the objective of their work. Nonetheless, SCG could also be used directly for 2,3-BD production, upgrading this lignocellulose-rich waste, since 2,3-BD has several valuable coproducts and can be converted to polymer precursors, as will be described next.

**Table 1:** Examples of the lignocellulosic biomasses used for 2,3-BD production.

Lignocellulosic Biomass	Production Method	Type of Sugar	2,3-BD Yield	References
Mixed biomass	Hydrolyses and flask fermentation by *S. cerevisiae*	Xylose	0.27 g/g	[49]
Sorghum biomass and wood	Hydrolyses and shaken flask, followed by bioreactor fermentation by *B. licheniformis*	Glucose and Xylose	0.45 g/g 0.40 g/g	[50]
Corncob	Alkali pretreatment, hydrolyses, and batch/fed-batch fermentation by *E. cloacae*	Glucose and Xylose	0.42 g/g	[51]
Kenaf core	Calcium peroxide pretreatment, hydrolyses, and batch fermentation by *K. pneumoniae*	Glucose and Xylose	0.38 g/g	[52]
Sunflower and pine tree	Hydrolyses and shaken flask fermentation by *K. oxytoca*	Glucose, Xylose, Galactose, and Mannose	0.29 g/g 0.22 g/g	[53]
Sugar cane bagasse	Hydrolyses and fed-batch fermentation by *E. ludwigii*	Xylose	0.38 g/g	[54]
Brewer’s spent grain	Microwave-assisted alkali pretreatment, hydrolyses, and shaken flask fermentation by *E. ludwigii*	Glucose	0.48 g/g	[55]

#### 3.1.1. Conversion of 2,3-BD into Biopolymer Precursors

It is well established that 2,3-BD can produce polymer precursors and other derivatives, such as methyl ethyl ketone (MEK), 1,3-butadiene (BD), acetoin, diacetyl, 2,3-butanediol diester, and polyurethane precursor. From dehydration, 2,3-BD can be converted into 1,3-BD using different catalysts, which are commonly applied in elastomer production, such as styrene-butadiene rubbers (SBR) [42,46,56]. Nguyen et al. (2019) produced 1,3-BD through the one-step catalytic dehydration of 2,3-BD with a rare earth orthophosphate-based catalytic reaction. They showed that this process could reach the industrial scale, with an operating temperature of 300 °C. The results also show that it was possible to recover 58 wt.% of 1,3-BD with MEK and methyl propanal (MPA) as coproducts [57]. Sun et al. (2020) reviewed several works about the extraction of C4 alcohols from biomass to produce 1,3-BD. They concluded that one of the most efficient and competitive methods for recovering 1,3-BD is through 2,3-BD production, mainly from lignocellulosic biomasses [58].

MEK is also produced from the dehydration of 2,3-BD, a fuel additive that can be used in the polymer industry to produce resins, paints, and solvents. From polymerization, 2,3-BD can be used as a polyurethane precursor, i.e., for polyol and polymeric isocyanates [42,59].

#### 3.1.2. Valorization of the Coproducts from 2,3-Butanediol Synthesis

The microbial synthesis of 2,3-BD results in the formation of coproducts, such as ethanol, acetoin, acetate, lactate, succinate, and formate [41,45]. Rehman et al. (2021) produced 2,3-BD from pure glucose and xylose-oil-palm-derived fermentation using *K. pneumoniae*, and they identified the formation of small amounts of ethanol, lactic acid, and succinic acid [43]. Narisetty et al. (2021) synthesized 2,3-BD from sugarcane-bagasse-rich xylose with *E. ludwigii*, and they obtained acetic acid as a coproduct [54]. Liakou et al. (2018) used various fruits, such as plums, apples, and pears, as well as vegetables, such as broccoli, cabbage, lettuce, fresh beans, corn salad, carrots, peppers, and eggplants, to extract fructose, sucrose, glucose, xylose, galactose, and arabinose, which were fermented by *E. ludwigii* to produce succinic acid, ethanol, and lactic acid as coproducts from 2,3-BD synthesis [60]. Furthermore, all these coproducts from 2,3-BD synthesis are valuable compounds that can also be directly extracted from the fermentation of the lignocellulosic biomass.

Bioethanol, produced from biomass sources, is biodegradable and less polluting than conventional ethanol, and can be used as a fuel for transport. From ethanol dehydration, it is possible to produce ethylene with a low investment. Ethylene has applications in the polymer field, e.g., in biobased polyethylene [61]. Previous research shows that bioethanol could be effectively produced from lignocellulosic biomass fermented by *S. cerevisiae* [62] and *Antarctic psychrophilic* [63]. Mussatto et al. (2012) evaluated the ethanol production from SCG sugars (glucose, arabinose, galactose, and mannose) fermented with three different strains (*S. cerevisiae, Pichia stipitis,* and *Kluyveromyces fragilis*). The results show that *S. cerevisiae* was the most effective for ethanol production: 11.7 g/L with a yield of 0.26 g/g [64]. Rocha et al. (2014) produced ethanol from the hydrolysis of SCG oil-free extract by ultrasound-assisted extraction, fermented by *S. cerevisiae* [65]. In addition, SCG oil was used with brewer’s spent grain to produce high amounts of ethanol (30–55 wt.%), for which the authors evaluated the relation between the increasing yield (more than 100%, compared to SCG oil alone) and the decreasing cost (from EUR 9.31/kg of SCG oil, to EUR 3.89/kg of SCG/brewer’s spent grain) for the ethanol produced [66].

Acid compounds, such as succinic, acetic, and lactic acids, are usually found from the beginning until the end of the SCG fermentation process [67]. Succinic acid is largely used in the production of 1,4-butanediol, tetrahydrofuran, and biodegradable polymers, such as poly(ester amides) [68]. Acetic acid is applied in vinyl acetate production, which is of interest for the production of vinyl plastics, adhesives, textile, and latex paints [69]. It also works as a solvent for the precipitation polymerization of poly(divinylbenzene) (PDVB) [70]. Lactic acid has been used in the pharmaceutical, cosmetic, chemical, and food industries for several years. Today, its main application is for poly(lactic acid) (PLA) production, a well-known biodegradable and biocompatible polyester [71,72].

Some of the coproducts from SCG fermentation are listed in Table 2. Liu et al. (2021) investigated the fermentation of SCG hydrolysates by *S. cerevisiae* and *Lachancea thermotolerans,* with and without yeast extracts. The results show that both strains produced succinic acid, acetic acid, lactic acid, and volatile compounds, but that the addition of yeast extract improved the succinic acid yield [12]. In another work, Liu et al. (2021) used SCG hydrolysates fermented by *Oenococcus oeni* and *L. thermotolerans* to produce volatile and nonvolatile compounds. The authors identified succinic, acetic, and lactic acids on SCG hydrolysates, before and after the fermentation process, with higher yields at the end of the fermentation process [73]. The specific production of lactic acid from SCG hydrolysates fermented by *Lactobacillus rhamnosus* was conducted and was aimed at industrial and economic demands [10]. Moreover, it was reported that 1000 g of SCG could produce about 100 g of lactic acid through the fermentation of SCG hydrolysates by *Lactobacillus brevis* and *Lactobacillus parabuchneri* [74]. Another work investigated lactic acid production via acid-pretreated SCG and washed pretreated SCG fermented by *S. cerevisiae*. The lactic acid yield was four times higher than the SCG pretreated with acid [75].

### 3.2. Polyhydroxyalkanoate (PHA) Synthesis

PHA is a bioplastic that is produced as an alternative to petroleum-based polymers with respect to sustainable industrial development [76]. A microbial fermentation process is used for the synthesis of this polyester, but it has a higher cost than petroleum-based polymers (USD 3.50/kg compared to USD 1.30/kg). Several efforts have been devoted to the use of waste biomass as a substrate source for PHA production, as the substrate represents around 28–50% of the entire PHA production costs [77,78]. Since PHA is biodegradable and biocompatible, it is used in several fields, such as in the production of biomedical devices (surgical pins and bone screws), as well as in electronics, construction, and the automotive industry, and in packaging, wrapping films, and bottles [77].

**Figure 3:**
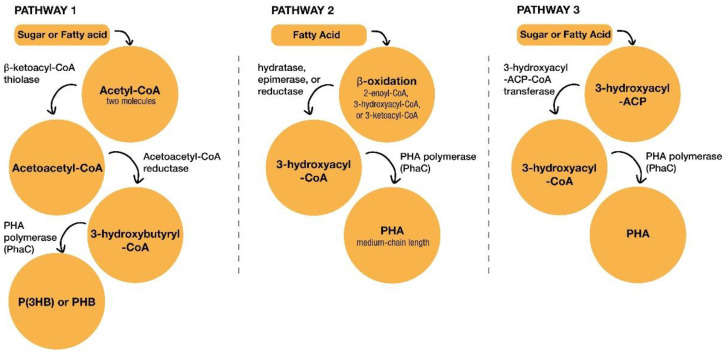
The three pathways leading to PHA synthesis.

PHA can be found with short-chain lengths (3–5 carbon atoms) and medium-chain lengths (6–14 carbon atoms) [77,79]. The former is more crystalline and has thermoplastic properties, while the latter has more elastomeric properties. Short-chain-length PHA is the most used and competes with poly(lactic acid) (PLA) in several fields, such as food packaging. Its functional groups can be replaced by 3-hydroxybutyrate or 3-hydroxyvalerate, forming poly(3-hydroxybutyrate) P(3HB), poly(4-hydroxybutyrate) P(4HB), poly(3-hydroxyvalerate) P(3HV), and a copolymer of P(3HB–co–3HV) [77,79]. PHA can be synthesized via three pathways: the acetyl-CoA to 3-hydroxybutyryl-CoA pathway; the fatty acid degradation by β-oxidation pathway; and the fatty acid synthesis pathway (Figure 3). Sugars (glucose, sucrose, and saccharose) and fatty acids (myristic, palmitic, and oleic) can be used as substrates for PHA production [77]. In the first pathway, the sugars or fatty acids are converted to acetyl-CoA. Two molecules of acetyl-CoA are converted to acetoacetyl-CoA by β-ketoacyl-CoA thiolase. In the next stage, acetoacetyl-CoA is reduced to 3-hydroxybutyryl-CoA by acetoacetyl-CoA reductase. Finally, PHB or P(3HB) is produced by polymerizing 3-hydroxybutyryl-CoA with PHA polymerase (PhaC). The second pathway starts from the fatty acid and β–oxidation cycle, which can be performed through 2-enoyl-CoA by hydratase, through 3-hydroxyacyl-CoA by epimerase, or through 3-ketoacyl-CoA by reductase. Then, 3-hydroxyacyl-CoA is obtained and converted to the PHA medium-chain length by PHA polymerase (PHaC). In the third pathway, sugars or fatty acids are converted to 3-hydroxyacyl-ACP. Through the enzyme, 3-hydroxyacyl-ACP-CoA transferase, 3-hydroxyacyl-ACP is converted to 3-hydroxyacyl-CoA, and this is followed by the synthesis of PHA polymerized by PHAC [11,76]. It should be noted that PHA production can be improved with an enhanced microbial fermentation process using specific conditions, such as fed-batch fermentation, the feast–famine strategy, solid-state fermentation, and continuous fermentation [79].

The 3-hydroxybutyrate (PHB) homopolymer can be produced from SCG oil extracted by supercritical carbon dioxide and fermented by *Cupriavidus necator*. The PHB yield can reach 0.77 kg per kg of SCG oil. Moreover, the mechanical properties of PHB films show a tensile strength of 16.0 MPa, a Young’s modulus of 1.0 GPa, and an elongation at break of 1.3% [80]. Kovalcik et al. (2018) produced PHB after the detoxification of SCG hydrolysates (the extracts of coffee oil and phenolics) by *Halomonas halophila* fermentation [81]. Obruca et al. (2014) also tested the detoxification of SCG hydrolysates to produce PHA by *Burkholderia cepacia*. It was found that polyphenol removal by ethanol was the most suitable process since it improved the PHA yield up to 25%. Therefore, copolymers of 3-hydroxybutyrate and 3-hydroxyvalerate were produced [82]. Although several works report using cellulose and hemicellulose sugars from lignocellulosic waste for PHA production [78,79,83], lignin is also an effective substrate source since acetyl-CoA can be produced from these aromatic compounds, followed by fatty acid synthesis to produce PHA [77,84].

**Figure 4:**
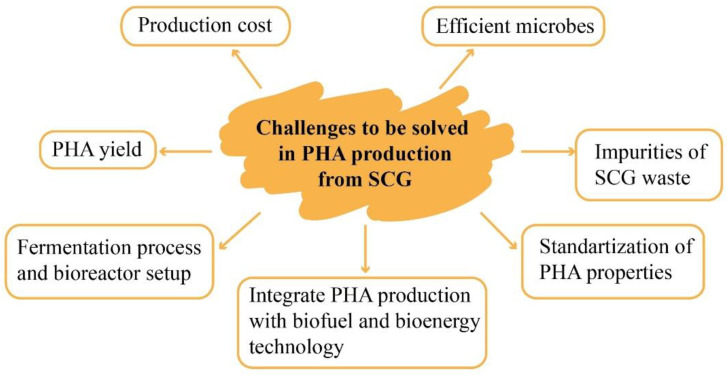
Challenges related to PHA production from SCG.

Several works focus on producing higher yields of PHA from waste biomass. However, effective extraction methods and the quality of the biopolymer properties require more attention. Moreover, waste biomass is not only used to reduce the PHA production costs; it is also the key to a circular economy because it closes the loop of material consumption [85]. The challenges in producing PHA from SCG are highlighted in Figure 4, while PHA production from SCG oil will be detailed in Section 4 with regard to the extraction and application of SCG oil.

### 3.3. Other Biopolymer Precursors

Other polymers can be produced from sugars, such as cyclic monomers, with or without the functional groups of the original sugar structure [86], and synthetic polymers, with sugar units integrated into their main chains [87]. Lactic acid is a cyclic monomer generally produced from glucose and sucrose fermentation, which can be polymerized into poly(lactic acid) (PLA) through polycondensation or ring-opening polymerization (ROP). Another example is the cyclic monomer, ε-caprolactone (ε-CL), produced from fructose, glucose, or mannose by 5-(hydroxymethyl)furfural. ε-CL can react with ammonia to synthesize Nylon-6 (ε-caprolactam), or by ROP to produce poly(ε-caprolactone) (ε-PCL) [86]. Polyesters, polycarbonates, polyamides, polyesteramides, polyurethanes, and polyureas can all be synthesized using sugar-based monomers, such as alditols, aldonic acids and lactones, aldaric acids (polyamides and polyesters), and amino sugars (polyamides, polyurethanes, and polyureas) [87]. Moreover, some sugar-based polymers are biodegradable, biocompatible, and nonimmunogenic. This is why they are mostly used in biomedical applications [88].

It was reported that aliphatic polyesters could be produced from sugar-based bicyclic alditol monomers from D-glucose (glux-diol and isosorbide) and D-mannose (manx-diol) by polycondensation. Nevertheless, glux-diol is the most suitable monomer in terms of the molecular weight (46,500 g/mol), the higher glass transition temperature (more than 100% increasing), and the higher mechanical properties (a 300% increase in tensile strength) [89]. Moreover, polycarbonate was effectively synthesized from the cyclic carbonate monomer obtained from D-mannose, showing good thermal properties for biomedical applications [90]. Polyols were also effectively produced from sugars and were used in polyurethane reactions. Fructose presented the most appropriate chemical properties for a fructose–isocyanate reaction, and resulted in a stable and low-shrinkage sugar-based polyurethane [91]. All these works confirm the possibility of producing a sugar-based polymer from different lignocellulosic sources. In particular, SCG were used in polyol production through the acid-liquefaction process, followed by polyurethane (PU) production. It should be noted that the optimal parameters for polyol acid liquefaction from SCG were 160 °C, 4 wt.% of sulfuric acid, and a reaction time of 80 min, which resulted in a yield of 70 wt.% of polyol [7]. Another work produced 74 wt.% of polyol from SCG, and then polymerized a PU foam (10–50 wt.% of SCG polyol) with a dodecahedron-cell structure and undamaged cell windows, similar to conventional PU foams for thermal insulation [92]. PU foam from SCG polyol has already been tested for thermal insulation applications and it demonstrates optimum thermal properties. Moreover, the foam exhibited great elasticity properties, since foams that were compressed to up to 70% could return to their initial state [93]. Polyols from SCG were also polymerized into PU foams for sound-absorption applications. The results show sound-absorption coefficient improvements at low and high frequencies, compared to the foams derived from glycerol, because of the increase in the cells size and the low stiffness of SCG, respectively [94].

Finally, a biofilm from the sugars of SCG was reported, using SCG galactomannans treated by alkaline and enzymatic treatments. The mechanical results show tensile strengths of 0.30 MPa and 0.21 MPa, Young’s moduli of 4.8 MPa and 8.5 MPa, and elongations at break of 13.2% and 5.26%, for the alkaline treatment and the enzymatic treatment, respectively. The water vapor permeability was also investigated, resulting in 2.3 g/m s Pa for the alkaline treatment, and 9.35 g/m s Pa for the enzymatic treatment. The authors conclude that both biofilms can be used for food packaging, vibration damping, and gas separation membranes [95].

## 4. SCG Oil

SCG oil has been extracted for applications in several fields, such as biodiesel production, polymer production, and cosmetics [81]. Coffee oil (corresponding to 7–21 wt.% of SCG) is rich in lipids/fatty acids (linoleic, palmitic, oleic, polyphenolic, etc.) and other valuable compounds, such as tocopherol and polyphenolic compounds. The oil can be recovered by solvent extraction using hexane, ethanol, and methanol, or by supercritical conditions with CO_2_. Supercritical CO_2_ extraction is more sustainable than solvent extraction and it can recover a higher oil yield (15–20%, instead of 6–27%) [13]. The combination of the two-phase solvent (methanol and hexane), assisted with ultrasound, can more effectively extract the coffee oil (higher yield), compared to the solvent extraction alone [96]. Nonthermal plasma technology and ultrasound-assisted methods were used with Soxhlet extraction (solvent) to investigate their influence on the oil yields. The results show that plasma technology recovered the highest amount of oil (19%), compared to ultrasound (14%) and Soxhlet extraction alone (9.4%) [97]. Couto et al. (2009) obtained coffee oil by supercritical CO_2_ under different temperatures, pressures, and time conditions. It was shown that 50 °C, 25 MPa, and 3 h were the optimal parameters, leading to a yield of 15%, which represented 85% of the total amount of oil [98]. Araújo et al. (2019) used ethanol as a solvent with supercritical CO_2_ to enhance coffee oil extraction. The results show a yield of 16%, but a shorter extraction time and a lower amount of organic solvent were used, compared to supercritical CO_2_ alone [99].

Several efforts have been made to produce PHA from SCG oil and to optimize the process to increase the PHA yield in a shorter time. One study shows that the homopolymer, 3-hydroxybutyrate, could be obtained from SCG oil extracted by supercritical CO_2_ [80]. The optimal parameters of the oil extraction were: 50 °C; 25 MPa; a solvent flow rate of 10 kg/h; a CO_2_:coffee mass ratio of 35:1; and 1.5 h of extraction time. These conditions enabled the recovery of 90% of the total coffee oil. Then, the coffee oil was fermented using *Cupriavidus necator* in a batch reactor, allowing a PHA conversion of 78% (*w*/*w*), with a molecular weight of 2.34 × 10^5^ g/mol, a melting temperature of 172 °C, a glass transition of 8 °C, and a crystallinity degree of 58% [80].

Obruca et al. (2014) used SCG oil fermented by *C. necator* in a batch and fed-batch reactor for PHB production. The fed-batch mode produced a higher PHB yield (49.4 g/L vs. 26.5 g/L), increased productivity (1.33 g/h vs. 0.66 g/h), and it produced a higher molecular mass (4.74 × 10^5^ vs. 4.27 × 10^5^ g/mol), compared to the batch mode. The authors also discuss the advantages of reusing SCG as a carbohydrate source for PHB production, since millions of tons of waste with toxic compounds are discarded in the environment (caffeine and tannins) [100].

Bhatia et al. (2018) obtained coffee oil from four different solvents (hexane, isobutanol, methanol, and ethanol), and fermented the solvent with the highest oil yield (coffee oil from hexane) with *Ralstonia eutropha* to produce a copolymer of poly(3-hydroxybutyrate-co-3-hydroxyhexanoate) (P(HB-co-HHx)). Using a β-oxidation pathway and coffee oil as a carbon source, it was possible to produce the copolymer precursor (HB-co-HHx). Then, 69% (*w*/*w*) of P(HB-co-HHx) was obtained, with 78 mol% of HB, and 22 mol% of HHx [101].

Ingram and Winterburn (2021) used coffee oil and sunflower seed oil as carbon sources for poly(3-hydroxybutyrate-co-3-hydroxyvalerate) (P(3HB-co-3HV)) production, fermented by *C. necator*. After 72 h, the coffee oil and the sunflower oil were produced, with 89% (*w*/*w*) and 88% (*w*/*w*) P(3HB-co-3HV) yields, respectively, showing that P(3HB-co-3HV) can be produced without any traditional 3HV precursor. The authors claim that it was the first time that P(3HB-co-3HV) was produced via *C. necator* from coffee oil, which will have a large influence on future works in this field [102].

## 5. SCG Polymer Composites

Polymers may be classified as thermoplastics, thermosets, and elastomers, on the basis of their responses to temperature. Above their melting temperature, thermoplastics become soft, presenting a fluid-like behavior, and they can then be molded. However, heating a thermoset eventually leads to its degradation [103]. Elastomers can have thermoplastic characteristics, or chains developing a network via covalent crosslinks, formed in a separate post-polymerization stage known as “vulcanization” [104]. In this study, some of the polymers used as matrices for the composites of SCG are presented and discussed.

### 5.1. Polythylene (PE) Composites

The most common synthetic polymer matrices filled with SCG are polyethylene (PE) and polypropylene (PP), because of their widespread applications in packaging, their low costs, and their overall good properties [2,105]. PE can be classified into several types, but high-density polyethylene (HDPE) and low-density polyethylene (LDPE) are the main commercial ones. Worldwide, PE is one of the most widely used thermoplastic polyolefins for blow and injection molding. Because of its high toughness, its ease of processing, its low electrical conductivity, and its chemical inertness, PE is used in different applications, such as in pipes, sheets, containers, and other similar products. Compared to LDPE, HDPE presents excellent impact strength and electrical insulation properties [106,107,108]. As a thermoplastic matrix for composites, HDPE can be found in packaging and automotive parts, as well as in biomedical and space applications [107,109].

Mendes et al. (2021) obtained HDPE/SCG composites (10–30 wt.%) via extrusion and injection molding. They reported that the incorporation of SCG particles in the HDPE preserved, or improved, the physicomechanical properties of the composites, without treatment or the use of a coupling agent. Adding SCG fibers increased the stiffness (elastic modulus) by 49% but decreased the tensile strength by 21%. Nevertheless, a 13% increase in the elasticity was observed with 10 wt.% of SCG. The incorporation of SCG did not modify the thermal properties, so the same processing conditions were used as in the neat HDPE matrix [110].

Alkali-treated SCG were used as fillers for the oxo-biodegradable HDPE composites, using different volume fractions (5, 10, 15, and 20%). The alkaline-treated SCG composites presented better structural, thermal, and mechanical properties than the untreated ones. The FTIR spectra show that the alkali treatment eliminated amorphous contents and impurities, improving the filler/matrix interface adhesion, as is confirmed by the SEM images. The degree of crystallinity increased by 5% after the treatment, and the thermal stabilities for both the untreated and treated composites were similar. In terms of the mechanical properties, at 10 wt.% of SCG, improvements of 25% for the tensile strength, and 24% for the tensile modulus, were observed, compared to the untreated composite. Using 15 wt.% of SCG led to a 6% improvement in the impact resistance. The authors indicate that this composite has high potential for several engineering applications, such as automotive, packaging, and lightweight furniture applications [111].

Cestari and Mendes (2013) elaborated the HDPE/SCG composites using four different types of SCG (integral, extracted, large-size, and small-size) in order to study the effects of the particle sizes and the soluble extraction on HDPE properties. The composites were prepared with 10 wt.% of filler. The results show that integral SCG presented similar properties to the small-size sample, and that it was superior to the extracted sample. The thermal properties show that no degradation occurred for the processing temperature range investigated (160–190 °C) for HDPE. HDPE/integral SCG and HDPE/small-size SCG composites degraded similarly, at higher temperatures than HDPE/extracted SCG and HDPE/large SCG. The melting temperature of all of the composites was similar to the neat polymer (134 °C). Except for the large SCG composites, a slight reduction in crystallinity was observed [112].

The effect of the SCG content (0 to 60 wt.%) on the crystallization kinetics of recycled HDPE composites was studied using differential scanning calorimetry (DSC). The results show that no degradation occurred within the processing temperature range (from −220 °C to 178 °C) of recycled HDPE. Moreover, the melting temperature of the composites was similar to the neat polymer (131 °C) [113].

Recycled HDPE compounds were elaborated from 0 to 60 wt.% of SCG using an extruder, and the samples were injection molded. A thermal analysis shows that the composites degraded in two steps. The first degradation step occurred at around 250 °C, and it was followed by another step at around 350 °C. The DSC curves show that the melting temperature of the composites was similar to that of the recycled HDPE, which presented a second peak at around 160 °C, which was probably due to the presence of PP as a typical contamination. The compressive moduli of the composites were similar to the neat polymer. The composite with 30 wt.% of SCG had the same compressive modulus as the neat polymer. However, the composites with 40 and 50 wt.% fillers had 11% lower compressive moduli, while at a 60 wt.%, the loss was 29% [114].

Biochar from SCG pyrolysis was used as a filler for the HDPE composites produced via melt mixing. A rheological characterization was performed for different flow fields (linear and nonlinear dynamic shear flows). A decrease in the relaxation dynamics of HDPE macromolecules was observed, which was due to the porous structure of the filler. Stress relaxation measurements revealed pseudo-solid-like behavior for the composites containing high amounts of filler. The biochar improved the thermo-oxidative stability of the composites and modified their melting enthalpies, which decreased the polymer crystallinity, compared to the neat matrix. The authors claim that this decrease in the polymer crystallinity was reported early in the literature because of the reduction in the polymer chain mobility in contact with the natural particle surface [115].

### 5.2. Polypropylene (PP) Composites

Polypropylene is a low-cost polymer and a well-known general-purpose commodity thermoplastic used in different applications, including in packaging, films, fibers, and automotive parts. PP can be used alone, or as a matrix for composites with different reinforcements (particles, fibers, etc.), especially biobased materials, such as wood [116], sugarcane bagasse [117], hemp [118], and rice straw [119]. Nevertheless, recent works report that SCG are a promising filler for PP composites, leading to different PP structures. It was reported that PP homopolymer and copolymer were successfully filled with 10, 20, and 30 wt.% of SCG. Overall, the mechanical properties decreased with the increasing SCG content, compared to the neat PP. However, the impact strength was improved by 77% (10 wt.% SCG), compared to the PP homopolymer. The authors conclude that SCG must have a surface treatment in order to improve the interfacial adhesion with the matrix and to improve the tensile strength for future works. These composites, based on SCG and PP homopolymer, could be used as substitutes for virgin PP in some applications, where the impact strength and the sustainable principles are needed [16].

PP composites can be improved by using treated fillers to increase the interfacial adhesion [16,120]. Coupling agents are generally used to create a chemical bond between the matrix and the reinforcement. The most common ones are based on maleic anhydride-grafted polypropylene (MAPP), and they yield effective results. However, a direct surface treatment of the fiber can be performed using palmitoyl chloride, silane, and MAPP to improve the properties of PP reinforced with SCG (20 wt.%). The flexural properties of the composite were not affected by either treatment (palmitoyl chloride and silane) or MAPP (2 wt.%). However, palmitoyl led to a significant improvement (54%) in the impact strength. The thermal stability of the untreated SCG composite was higher than the neat PP, but the addition of MAPP and the surface treatments decreased it. Moreover, palmitoyl provided an effective hydrophobic behavior and showed a better SCG particle dispersion in the matrix, with the authors of the study concluding that palmitoyl-chloride-modified SCG was the best filler studied (20 wt.% SCG) [17]. MAPP (1, 3, and 5 wt.%) was also reported with PP homopolymer and copolymer, reinforced with 15 wt.% of SCG [121]. The PP copolymer, with and without MAPP, presented the best mechanical results, which were related to a better interaction between the chemical structure of the copolymer (propylene and ethylene) with the MAPP and the OH groups of the filler surface. The MAPP content was found to directly affect the mechanical properties, but 3 wt.% of MAPP showed a balance between all the results [121]. The other coupling agents investigated were silane and styrene–ethylene–butene–styrene grafted with maleic anhydride (SEBS-g-MA) for the PP composites filled with 15 wt.% of SCG. The coupling agents were compared with the bleached SCG to enhance the interfacial adhesion between the matrix/filler by removing the amorphous components, which leads to an improvement in the surface roughness of the filler, for which, in this case, bleached SCG caused no significant mechanical properties (less than 10%). Both coupling agents had good mechanical properties, but SEBS-g-MA was the only one that slightly improved the tensile strength (6%) without losing ductility, and while maintaining rigidity (Young’s modulus) [18].

Wu et al. (2016) report that the oil extracted from SCG could improve the properties of PP composites. The oil extraction was performed by ultrasonication, followed by the production of composites with 40 wt.% of SCG, 2.5 wt.% of MAPP, and 2.5 wt.% of stearic acid. The composites with oil-extracted SCG presented lower water absorption, combined with higher mechanical strength (12%). These results validate that this additive is efficient at improving PP composites [120]. However, the oil-extracted SCG can also be used in other applications, such as biopolymer or biodiesel production [122]. Another interesting study was reported on SCG and coffee chaff as fillers for PP composites. Although SCG are more widely known and available, coffee chaff led to better thermal stability (an increase of 35 °C), better tensile (16%) and flexural (21%) strengths, as well as better tensile (43%) and flexural (52%) moduli. However, the coffee chaff produced a more brittle composite (lower elongation at yield (45%) and at break (55%), with a similar impact strength). Furthermore, coffee chaff has a dense fibrous morphology, while SCG present a granular porous morphology that is directly related to the better mechanical improvement of coffee chaff [123].

One of the most significant advantages of PP is its thermoplastic behavior, allowing for the possibility of using a recycled resin as the matrix to produce composites, thereby developing a more environmentally friendly material, with properties similar to those of virgin PP. Recycled PP (rPP) was applied in several works, with different fibers [124,125,126]. However, only one work was found on combining rPP with SCG, where the rPP was obtained from waste espresso coffee capsules and was reinforced with 20 and 30 wt.% of espresso SCG, with and without MAPP (10 wt.% of the reinforcement amount). The mechanical properties and the thermal stability results show that the composites based on rPP had properties similar to those of the virgin PP. Moreover, the effect of MAPP led to increases in the tensile strength (18%) and the Young’s modulus (20%), but to decreases in the elongation at break (more than 100%) and the impact strength (23%). However, these composites were developed for a specific application on home composters. The authors conclude that the composite with 30 wt.% of SCG and without MAPP was the most suitable for this application, which was aiming for an environmentally friendly product [127].

### 5.3. Polyurethane (PU) Composites

Polyurethane is a versatile polymer that was created to replace rubbers by reacting a diisocyanate with a polyol. It has been used in several fields, such as in insulators, rigid foams, coating, adhesives, and elastomers [128]. Because of environmental concerns, some efforts are devoted to producing polyol from a natural source, and to producing PU composites with natural or waste-based fillers. So far, a very limited number of works are available on using SCG as a filler in PU composites.

Funabashi et al. (2003) compared different fillers in rigid PU foam composites, including SCG [129]. Hatakeyama and Hatakeyama (2010) produced PU from lignin polyol filled with SCG (50–80 wt.%). The results show increases in the flexural strength and the modulus, as the amount of filler increased with the constant density [130]. More recently, SCG (10–40 wt.%) were added to a viscoelastic PU foam composite, leading to a reduction in the foam growth time (11% for 10 wt.% SCG), and an increase in the foam density with increasing filler content (24% for 30 wt.% of SCG). The compression tests after 75% and 90% of the original deformation show that PU/SCG composites presented lower values of permanent deformation (about 2–3%), compared to neat PU (10% and 85%), which were below the acceptable limit of 10% [15].

### 5.4. Poly(Lactic Acid) (PLA) Composites

Biodegradable polymers have been reported to be more costly. Therefore, PLA composites with lignocellulosic wastes have been produced to reduce the PLA content, consequently becoming more affordable for the different applications for replacing synthetic polymers. In the last ten years, several works have reported on SCG valorization as a filler for PLA composites to reduce the amount of SCG in the environment. Baek et al. (2013) studied the effect of the SCG content (10, 20, 30, and 40 wt.%) in PLA and 4,4-methylene diphenyl diisocyanate (MDI) as a coupling agent. The mechanical strength decreased with the increasing filler content, but MDI was shown to improve the values at 30 wt.% by creating an urethane bond between the PLA and SCG [131]. Furthermore, the tensile strength of the 30 wt.% of the PLA/SCG composite without MDI (27.5 MPa) [131] was slightly higher than for the 30 wt.% of the PP copolymer/SCG composite (26.0 MPa) [16]. This comparison between the PLA and PP composites highlights the possibility of using PLA as a substitute for synthetic polymers, without using a nonenvironmentally friendly coupling agent, such as MDI. Arrigo et al. (2020) investigated two alternative processing routes to produce PLA filled with SCG biochar (1, 2.5, 5, and 7.5 wt.%): melt mixing and solvent casting. Both processes successfully produced PLA/biochar composites. However, a rheological characterization study suggests a poor particle dispersion for the solvent casting process, while polymer degradation (lower molar mass) was observed for the melt mixing process because of the higher temperature [21].

The main advantage of PLA reinforced with natural materials is the biodegradability of both materials, resulting in environmentally friendly compounds with low carbon footprints and easy end-of-life disposal (composting). The biodegradation rate was investigated for PLA/SCG composites, with and without coupling agents. After 60 days of incubation, the results show that the PLA filled with 20 wt.% of SCG had a higher mass loss than the composites with a coupling agent and the neat PLA. This biodegradation rate change was supported by a microscopy analysis, which showed more disruptions with larger voids for the PLA/SCG without a coupling agent [23]. It was also reported that a photodegradation test could precede the biodegradation test, accelerating the degradation process in PLA/SCG composites. The results show a decrease in the crystallinity degree and impact strength, but an increase in the water absorption, compared to the biodegradation test alone. Moreover, SEM micrographs show that the photodegradation followed by biodegradation caused a roughening of the material surfaces, producing more cracks, voids, and erosions [20].

Some efforts have been devoted to the food packaging field, mainly through the development of PLA biofilm composites. SCG were reported to act as a plasticizer and a lubricant in biofilms. The results show a more flexible PLA behavior (higher elongation at break) after the addition of SCG [132]. It was also shown that SCG could act as an oxygen barrier in PLA reinforced with diatomite, improving their possible use as food packaging [133]. Songtipya et al. (2019) investigated the use of PLA with polybutylene adipate terephthalate (PBAT) and SCG for food packaging, using toluene 2,4-diisocyanate (TDI) as a coupling agent. The composite presented good mechanical properties, with overall migration values (0.03–0.28 mg/dm^2^) lower than 10 mg/dm^2^, which is the limit for chemical compounds on the surfaces of food packaging. This indicates that these PLA/SCG biofilms can be used for food packaging, with a minimum number of affinities among the chemical compounds of the polymer and the food [19].

Besides food packaging, PLA has been widely used as a filament for 3D printing. Recently, researchers were looking to produce lower-cost PLA filaments by using different fillers, such as lignocellulosic wastes. PLA filled with oil-extracted SCG was effectively produced, and they presented an important toughness increase (419%), with 20 wt.% of SCG, compared to the neat PLA filament, which also led to a higher impact strength [134]. In another work, SCG were decolorized by bleaching and were mixed with PLA to change the final printing color of the PLA/SCG filament from brown (SCG) to other colors, depending on the pigments used. The decolorized composites showed similar mechanical strengths to the neat PLA, resulting in colored filaments with high melt flows and good printing quality [135].

SCG oil was also proposed as a plasticizer for PLA composites, using 40 wt.% of recycled coffee cups (paper). The results show that the SCG oil plasticizer (30 wt.%) improved the hydrophobicity and decreased the brittle behavior of the composite (an 86% increase in the elongation at break). The authors state that this composite had balanced mechanical properties and a nontoxic behavior for several food applications, especially for the coffee beverage industry [136]. SCG were also investigated to produce luminescent quantum dots (QD) as a filler for PLA. A composite with 1 wt.% of QD showed good UV shielding and important transmission to the visible light, coupled with significantly improved mechanical properties, compared to the neat PLA, i.e., 69% and 67% increases in the tensile strength and the elastic modulus, respectively. The authors also propose the use of this material as a high-performance nanocomposite for applications involving transparency and UV protection [137]. Another interesting work reported good results by using PP and lignin as a compatibilizer for PLA/SCG composites. PP and lignin were combined to improve the mechanical, thermal, and morphological properties, compared to those of neat PP or lignin alone, and they showed some synergy and a compatibilization efficiency for PLA/SCG composites [138].

### 5.5. Poly(Butylene Adipate-Co-Terephthalate) (PBAT) Composites

PBAT is a biodegradable polymer that is mainly used in packaging and the biomedical fields. Because of its low thermomechanical properties and high production cost, PBAT was used as a matrix for lignocellulosic composites [139]. Coffee waste is a current material for PBAT composites filled with coffee husks [140,141] and coffee silver skins [142,143].

Moustafa et al. (2017) published two works on PBAT/SCG composites. The first one investigated the effect of a plasticizer (polyethylene glycol, PEG). The results show a good interaction between the SCG, PBAT, and the plasticizer, which led to a higher tensile strength, good SCG dispersion, higher hydrophobicity, and higher thermal stability [144]. In their second work, the effect of torrefied SCG on the hydrophobicity of PBAT composites was studied. It was shown that coffee torrefaction at 250 °C and 270 °C was efficient to improve the composite hydrophobicity by more than 20%, while also improving the tensile strength of 10 wt.% of torrefied SCG by 27% and 63%, compared to the neat PBAT and the nontorrefied SCG composite, respectively [145].

### 5.6. Polyvinyl Alcohol (PVA) Composites

Polyvinyl alcohol is a biodegradable, biocompatible, water-soluble, and hydrophilic synthetic polymer that is used in several fields, such as biomedical and food packaging [146]. PVA fully biodegradable composites filled with SCG have been reported in different studies, mainly for their adsorbent properties. Lessa et al. (2018) investigated PVA filled with SCG and chitosan to adsorb pharmaceutical contaminants from water. They observed a substantial improvement (from 10 to 40%) in the absorption properties with a 5 wt.% of SCG, compared to the PBAT/chitosan composite. It was also possible to remove acetylsalicylic acid, caffeine, acetaminophen, and metamizole from the water [147]. Another study developed PBAT composites on the basis of Fe_3_O_4_ for Pb(II) ion adsorption. The optimal adsorption conditions were at a pH of 5, 24 h of contact time, room temperature, and an SCG:Fe_3_O_4_ ratio of 4:1. It was shown that the PBAT/SCG/Fe_3_O_4_ maintained a 78% adsorption efficiency after five cycles [148]. Minh and Thuan (2021) also produced PBAT/SCG/Fe_3_O_4_ composites for the adsorption of methylene blue, congo red, and tannic acid from an aqueous solution. The composite was analyzed by adsorption kinetics and adsorption thermodynamics, and the results present a high adsorption capacity, as the process was characterized as spontaneous and endothermic, with a blend of physisorption (electrostatic interaction, internal and external pore diffusion) and chemisorption (load shared or transferred from the organic molecules to the surface functional groups of the sorbent to create a chemical bond) adsorption mechanisms [149].

Cellulose nanofibers extracted from SCG were also studied as fillers for PVA composites as a source of non-wood cellulose material [150]. It was reported that nanoparticles from SCG can be used as a filler for PVA composites, leading to a higher tensile strength (from 80 MPa to 125 MPa) and a better deodorization performance, which means the removal of compounds that causes undesirable odors (from 98.9% to 89.5%), compared to neat PVA and PVA/carbon black composites [151]. Another interesting work used antioxidants extracted from SCG and citric acid as an active compound for PVA/starch films. The authors report that these antioxidants were effective at improving the antioxidant and antimicrobial properties of the films for food packaging [152].

### 5.7. Epoxy Composites

The effect of the SCG addition (5, 10, 15, 20, 25, and 30 wt.%) on the mechanical properties of compression-molded epoxy composites was studied. Compared to the neat resin, better mechanical properties for SCG/epoxy composites were found, especially for 25 wt.% of SCG. The fracture toughness increased with the SCG content, and a uniform distribution in the epoxy matrix enabled good stress distribution and better interface bonding. The 30 wt.% of SCG presented highly reduced wettability, with the epoxy matrix leading to lower mechanical properties, e.g., a decrease of 50% in the ultimate stress, and a decrease of 23.8% in the toughness [153].

Chemically treated SCG with NaOH were mixed with an epoxy resin at different weight contents (30, 40, 50, and 60 wt.%) [154]. The “30 wt.% of SCG” composites presented the most suitable properties, with better compatibility, compared to the other concentrations. A tensile strength of 45 MPa, a flexural strength of 80 MPa, a compressive strength of 112 MPa, and an Izod impact strength of 8 kJ/m^2^ were reported. Their flame-retardant properties showed that the oxygen index was 20%, and the burning rate, according to the UL94HB, was 27 mm/min. Adding 30 wt.% of SCG with glass fiber led to the production of an epoxy hybrid composite. The morphological structure of the SCG/fiberglass/epoxy hybrid composite showed that the interface was strongly bonded and interactive, but no effect on the flame-retardant properties was observed, as only the epoxy resin and part of the SCG were burned, while the glass remained intact. The mechanical properties of the SCG/fiberglass/epoxy hybrid composite were similar to those of the epoxy-based composites reinforced with SCG alone.

Biochar derived from SCG (1 and 3 wt.%) was also used to obtain thermoset-based composites for 3D printing [155]. Their particles presented a nanostructured morphology. The rheological results show that the addition of SCG biochar increased the resin viscosity. Nevertheless, the 3D printed samples with lower SCG biochar contents (1 wt.%) had improved mechanical properties. The storage modulus increased by 27%, while the flexural modulus and strength increased by 55% and 43%, respectively. Unfortunately, adding 3 wt.% of SCG biochar substantially decreased the viscoelastic and flexural properties, which is due to agglomeration and the improper crosslinking between the chains. Moreover, SCG biochar (15 and 20 wt.%) was also blended with epoxy for electrical purposes [156]. The results show that composites with 20 wt.% of SCG biochar produced a higher electrical conductivity (four times) than carbon black composites, and a higher tensile strength (18%), compared to the carbon black composite and neat epoxy.

SCG oil was successfully extracted from SCG and then the extracted SCG were blended (10 wt.%) with an epoxy resin [157]. In general, the composites presented lower mechanical properties relative to the neat epoxy. However, improvements were observed for the extracted SCG composites, compared to the SCG composites: an increased tensile strength, from 20.9 to 23.4 MPa; an increased flexural modulus, from 2.09 to 3.02 GPa; and increased flexural strength, from 33.0 to 42.9 MPa. Another recent work extracted the oil from SCG to fill epoxy composites (35 wt.%), and they claimed that extracted SCG provided enhanced tensile strength (more than 200%) and toughness (more than 100%), compared to neat epoxy and the SCG composite. The curing kinetics were also investigated and showed that extracted SCG composites cured faster at room temperature [158].

A novel work used SCG treated with phosphorus as a flame retardant in epoxy composites (5, 15, and 30 wt.%) [159]. The results show that the sample with 30 wt.% of SCG presented a 40% decrease in the peak of the heat release rate compared to neat epoxy, which confirmed the flame-retardant behavior of the epoxy/SCG composite. Furthermore, the burning tests reveal the self-extinguishing behavior of this composite after 40 s of ignition, proving the efficiency of phosphorus treatment on SCG particles, which caused the flame-retardant behavior and formed a compact char.

### 5.8. Rubber Composites

For elastomers or rubbers as a matrix, SCG, PP, and PLA particles were used as fillers instead of the commonly used carbon black (CB) [160]. The effects of the filler additions on the vulcanization characteristics of the rubber compounds, as well as on the physical, mechanical, and dynamic mechanical properties, were analyzed. Compared to the reference sample, the minimum and maximum torque values of the PP, PLA, and SCG composites were lower, while the optimum vulcanization time for PP and SCG were slightly higher. This indicates that these alternative fillers lead to lower vulcanization rates. The tensile strengths of the PP and SCG composites were similar and slightly higher, compared to the reference. However, the hardness and storage moduli of the PLA and SCG composites decreased.

The properties of natural rubber (NR) filled with various amounts of SCG, and the surface treated by a silane coupling agent (TESPT) and liquid epoxidized natural rubber (LENR) was studied [161]. The incorporation of SCG resulted in a faster cure (50%) and a higher curing efficiency. However, it did not provide adequate reinforcement and it retarded the vulcanization process. The surface treatment improved the rubber properties, which is due to the better rubber–filler interaction and the higher cure. TESPT-SCG provided a composite with a higher crosslink density (21%), hardness (6%), and modulus (13%), compared to LENR-SCG, producing the highest mechanical properties, followed by LENR-SCG and untreated SCG, respectively.

A more recent work used SCG treated via pyrolysis as a filler for styrene–butadiene rubber (5, 10, 15, and 20 wt.%) [162]. The pyrolysis treatment (700 °C and 900 °C) decreased the average particle size of the SCG and improved the surface roughness, leading to a better interaction with the rubber than untreated SCG/rubber composites. The SCG treatment behaved as an activator for vulcanization, decreasing the cure time, increasing the crosslink density, and increasing the mechanical properties by 30% (the tensile strength and the modulus), compared to the untreated SCG/rubber composite.

## 6. SCG Reuse Routes

SCG have been widely used in other fields since their high amounts of generated waste are a concern for the environment; however, they are a nutrient-rich material, containing polysaccharides, lipids, proteins, and minerals. A recent review lists the potential SCG applications, which include their use as an antioxidant source, as well as in energy production, soil fertilizers, dietary fiber, adsorbents, biogas production, and microbial biotechnology [163]. In the present review, SCG reuse is focussed on two areas: biopolymer precursors and composite production. These two areas are different, but they can be complementary, since the composite’s matrix can be produced from a polymer based on SCG as a precursor.

As a raw material, SCG can be a filler for composites and a biofertilizer for the soil. Composites filled with SCG can initiate a new lifecycle as a plastic product, decreasing the amount of synthetic material, and they are biodegradable/compostable, especially when using polylactic acid (PLA) or polyhydroxyalkanoates (PHA) as matrices. Moreover, composites filled with lignocellulosic materials are being reported for food packaging [2,19,164], automotive parts [111,165], and household furniture [111,157].

On the other hand, SCG can be chemically treated to remove valuable compounds, generating other applications using their sugars or oil fractions. Sugars are directly related to the production of biopolymer precursors, as well as bioethanol, biogas, and biodiesel [13,22]. From coffee oil, it is possible to produce PHA, as well as pharmaceutical, food, and cosmetics products [13].

## 7. Conclusions

Several works report the use of SCG in the plastics field as an environmentally friendly material. As a starting point, several works investigate and discuss the possibility of extracting SCG polysaccharides, which can be fermented to produce polymer precursors, such as lactic acid and polyol, or that can directly produce a biopolymer, such as PHA. These works show that microbial fermentation is effective in extracting the main compounds from SCG. In particular, the oil fraction was found to be a valuable resource, not only for biofuel production, but also for PHA synthesis. SCG particles can also be included in different polymer matrices, such as PP, PE, PU, PLA, epoxy, and rubbers, to produce composites with suitable properties for numerous applications, such as food packaging, automotive parts, 3D printing, and UV shielding. Moreover, SCG can be used as a plasticizer.

Although a great deal of effort was devoted to combining SCG and different polymer matrices, some gaps in the literature were found and could be the subjects for future research. For example, 2,3-BD synthesis was reported as a valuable source of polymer precursors and coproducts, but nothing was found on the direct synthesis of 2,3-BD from SCG. Moreover, lactic acid, succinic acid, and other organic compounds could be directly extracted from SCG for novel applications in the biopolymer field, with the aim of developing new green polymers. Coffee oil, as a valuable fraction of SCG, could be further studied in the plastics field, not only for biopolymer synthesis, but also as biofillers for the production of composites and/or bioadditives to modify their properties. More works on polymer composites must be performed for a wider range of matrices, both synthetic and biobased. For example, PU made from SCG polyol, PLA made from SCG lactic acid, and PHA made from SCG could also be filled with SCG. In fact, nothing was found on PHA composites filled with SCG. Finally, recycled matrices should be further investigated, as well as the possibility of recycling these composites after their end-of-life.

## Figures and Tables

**Table 2:** Examples of coproducts from SCG fermentation.

Coproducts	Production Method	Yield ^1^	References
Bioethanol	Hydrolysis of SCG fermented by *S. cerevisiae*	0.26 g/g	[64]
Bioethanol	Hydrolysis of SCG oil extracted by ultrasound-assisted extraction fermented by *S. cerevisiae*	0.5 g/g	[65]
Bioethanol	Hydrolysis of SCG oil and brewer’s spent grain oil, extracted by Soxhlet extraction, fermented by *S. cerevisiae*	57.3%	[66]
Succinic acid, acetic acid, and lactic acid	Hydrolysis of SCG fermented by *S. cerevisiae* with yeast extract	2.6 g/L 0.8 g/L 0.2 g/L	[12]
Succinic acid, acetic acid, and lactic acid	Hydrolysis of SCG fermented by *O. oeni* coinoculated with *L. thermotolerans*	16.4 g/L 5.2 g/L 22.4 g/L	[73]
Lactic acid	Hydrolysis of SCG fermented by *L. rhamnosus*	98%	[10]
Lactic acid	Hydrolysis of alkali-treated SCG fermented by *L. brevis* (Lb) and *L. parabuchneri* (Lp)	40.1% (Lb) 55.8% (Lp)	[74]
Lactic acid	SCG pretreated with sulfuric acid whole slurry (s) and washed (w) and fermented by *S. cerevisiae*	11.2 g/L (s) 3.4 g/L (w)	[75]

^1^ When many different samples were found, only the highest yields are shown.
